# Supplementary material for: Heat adaptation of phage T7 under an extended genetic code
Source: Virus Evol. 2021 Dec 1;7(2):veab100. doi: 10.1093/ve/veab100 (PMC8923235; doi:10.1093/ve/veab100)
Supplement: veab100_Supp [file veab100_supp.zip › Sequencing_Analysis.zip!/Sequencing_Analysis/A3/evidence/JC_71.html]

BRESEQ :: Evidence


| New junction evidence | | | | | | | | | | | |
| --- | --- | --- | --- | --- | --- | --- | --- | --- | --- | --- | --- |
|  | | seq id | position | reads (cov) | reads (cov) | score | skew | freq | annotation | gene | product |
| \* | ? | NC\_001604.1 | = 13568 | 14 (0.010) | 134 (0.080) | 16/586 | 35.3 | 58.7% | intergenic (–/–) | *–/–* | –/– |
|  | ? | NC\_001604.1 | 14339 = | 175 (0.100) |  |  |  |  | intergenic (–/–) | *–/–* | –/– |
| Rejected: Coverage evenness skew score above cutoff. | | | | | | | | | | | |

Only 100 of 204 total aligned reads displayed.  

`AGGATTCTTGATGACTTTCCAGACGACTACGAGAAGTTTCGCTGGAGAGTCCCATTCTAATACGACTCACTAAAGGAGACACACCATGTTCAAACTGATTAAGAAGTTAGGCCAACTGCTGGTTCGTATGTACAACGTGGAAGCCAAGCGACTGAACGATGAGGCTCGTAAAGAGGCCACACAGTCACGCGCTCTGGCGATTCGCTCCAACGAACTGGCTGACAGTGCATCCACTAAAGTTACCGAGGCTGCCCGTGTGGCAAACCAAGCTCAACAGCTTTCCAAATTCTTTGAGTAATC‑AA‑‑‑‑‑‑‑‑‑‑‑‑‑‑‑‑‑‑‑‑‑‑‑‑‑‑‑‑‑‑‑‑‑‑‑‑‑‑‑‑‑‑‑‑‑‑‑‑‑‑‑‑‑‑‑‑‑‑‑‑‑‑‑‑‑‑‑‑‑‑‑‑‑‑‑‑‑‑‑‑‑‑‑‑‑‑‑‑‑‑‑‑‑‑‑‑‑‑‑‑‑‑‑‑‑‑‑‑‑‑‑‑‑‑‑‑‑‑‑‑‑‑‑‑‑‑‑‑‑‑‑‑‑‑‑‑‑‑‑‑‑‑‑‑‑‑‑‑‑‑‑‑‑‑‑‑‑‑‑‑‑‑‑‑‑‑‑‑‑‑‑‑‑‑‑‑‑‑‑‑‑‑‑‑‑‑‑‑‑‑‑‑‑‑‑‑‑‑‑‑‑‑‑‑‑‑‑‑‑‑‑‑‑‑‑‑‑‑‑‑‑‑‑‑‑‑‑‑‑‑‑‑‑‑‑‑‑‑‑‑‑‑‑‑‑‑‑‑‑‑‑‑‑‑‑‑‑‑‑‑‑‑‑‑‑‑‑‑‑‑‑‑‑‑‑‑‑‑‑‑‑‑‑‑‑‑‑‑‑‑‑‑‑‑‑‑‑‑‑  >  NC_001604.1/13267‑13568`  
`‑‑‑‑‑‑‑‑‑‑‑‑‑‑‑‑‑‑‑‑‑‑‑‑‑‑‑‑‑‑‑‑‑‑‑‑‑‑‑‑‑‑‑‑‑‑‑‑‑‑‑‑‑‑‑‑‑‑‑‑‑‑‑‑‑‑‑‑‑‑‑‑‑‑‑‑‑‑‑‑‑‑‑‑‑‑‑‑‑‑‑‑‑‑‑‑‑‑‑‑‑‑‑‑‑‑‑‑‑‑‑‑‑‑‑‑‑‑‑‑‑‑‑‑‑‑‑‑‑‑‑‑‑‑‑‑‑‑‑‑‑‑‑‑‑‑‑‑‑‑‑‑‑‑‑‑‑‑‑‑‑‑‑‑‑‑‑‑‑‑‑‑‑‑‑‑‑‑‑‑‑‑‑‑‑‑‑‑‑‑‑‑‑‑‑‑‑‑‑‑‑‑‑‑‑‑‑‑‑‑‑‑‑‑‑‑‑‑‑‑‑‑‑‑‑‑‑‑‑‑‑‑‑‑‑‑‑‑‑‑‑‑‑‑‑‑‑‑‑‑‑‑‑‑‑‑‑‑‑‑‑‑‑‑‑‑‑‑‑‑‑‑‑‑‑‑‑‑‑‑‑‑‑‑‑‑‑‑‑‑‑‑‑‑‑taatc‑aaTAGGAGAAATCAATATGATCGTTTCTGACATCGAAGCTAACGCCCTCTTAGAGAGCGTCACTAAGTTCCACTGCGGGGTTATCTACGACTACTCCACCGCTGAGTACGTAAGCTACCGTCCGAGTGACTTCGGTGCGTATCTGGATGCGCTGGAAGCCGAGGTTGCACGAGGCGGTCTTATTGTGTTCCACAACGGTCACAAGTATGACGTTCCTGCATTGACCAAACTGGCAAAGTTGCAATTGAACCGAGAGTTCCACCTTCCTCGTGAGAACTGTATTGACACCCTTGTGTTGTCA  >  NC_001604.1/14339‑14637`  

                                                                                                                                                                                                                                                                                                                                                                                                                                                                                                                                                                                                                            
`AGGATTATTGATGACTTTCCAGACGACTACGAGAAGTTTCGCTGGAGAGTCACATTCTAATACGACTCACTAAAGGAGACACACCATGTTCAAACTGATTAAGAAGTTAGGCCAACTGCTGGTTCGTATGTACAACGTGGAAGCCAAGCGACTGAACGATGAGGCTCGTAAAGAGGCCACACAGTCACGCGCTCTGGCGATTCGCTCCAACGAACTGGCTTACAGTGCATCCACTAAAGTTACCGAGGCTGCCCGTGTGGCAAACCAAGCTCAACAGCTTTCCAAATTCTTTGAGTAATC‑A                                                                                                                                                                                                                                                                                                              <  1:287148/301‑1`  
      `CTTGATGACTTTCCAGACGACTACGAGAAGTTTCGCTGGAGAGTCCCATTCTAATACGACTCACTAAAGGAGACACACCATGTTCAAACTGATTAAGAAGTTAGGCCAACTGCTGGTTCGTATGTACAACGTGGAAGCCAAGCGACTGAACGATGAGGCTCGTAAAGAGGCCACACAGTCACGCGCTCTGGCGATTCGCTCCAACGAACTGGCTGACAGTGCATCCACTAAAGTTACCGAGGCTGCCCGTGTGGCAAACCACGCTCAACAGCTTTCCAAATTCTTTGAGTAATC‑Actgtct                                                                                                                                                                                                                                                                                                        >  1:291429/1‑295`  
                           `aACGAGAAGTTTCGCTGGAGAGTCCAATTCTAATACGACTCACTAAAGGAGACACACCATGTTCAAACTGATTAAGAAGTTAGGCAAACTGCTGGTTCGTATGTACAACGTGGAAGCCAAGCGACTGAACGATGAGGCTCGTAAAGAGGCCACACAGTCACGCGCTCTGGCGATTCGCTCCAACGAACTGGCTGACAGTGCATCCACTAAAGTTACCGAGGCTGCCCGTGTGGCAAACCAAGCGCAACAGCTTTCCAAATTCTTTGAGTAATCAAACAGGAGAAATCAATATGATCGTTTC                                                                                                                                                                                                                                                                                    <  1:138859/300‑1`  
                                        `GCTGGAGAGTCCCATTCTAATACGACTCACTAAAGGAGACACACCATGTTCAAACTGATTAAGAAGTTAGGCCAACTGCTGGTTCGTATGTACAACGTGGAAGCCAAGCGACTGAACGATGAGGCTCGTAAAGAGGCCACACAGCCACGCGCTCTGGCGATTCGCTCCAACGAACTGGCTGACAGTGCATCCACTAAAGTTACCGAGGCTGCCCATGTGGCAAACCAAGCTCAACAGCTTTCCAAATTCTTTGAGTAATCAAACAGGAGAAACCATTATGATCGTTTCTGACATCGAAGCT                                                                                                                                                                                                                                                                       <  1:21344/301‑1`  
                                                `GTCCCATTCTAATACGACTCACTAAAGGAGACACACCATGTTCAAACTGATTAAGAAGTTAGGCCAACTGCTGGTTCGTATGTACAACGTGGAAGCCAAGCGACTGAACGATGAGGCTCGTAAAGAGGCCACACAGTCACGCGCTCTGGCGATTCGCTCCAACGAACTGGCTGACAGTGCATCCACTACAGTTACCGAGGCTGCCCGTGTGGCAAACCAAGCTCAACAGCTTTCCAAATTCTTTGAGTAATCAAACAGGCGAAATCAATATGATCGTTTCTGACATCGAAGCTAACGCCCT                                                                                                                                                                                                                                                               >  1:226920/1‑301`  
                                                `GTCCCATTCTAATACGACTCAATAAAGGAGACACACCATGTTCAAACTGATTAAGAAGTTAGGCCAACTGCTGGTTCGTATGTACAACGTGGAAGCCAAGCGACTGAACGATGAGGCTCGTAAAGAGGCCACACAGTCACGCGCTCTGGCGATTCGCTCCAACGAACTGGCTGACAGTGCATCCACTAAAGTTACCGAGGCTGCCCGTGTGGCAAACCAAGCTCAACAGCTTTCCAAATTCTTTGAGTAATCAAACAGGAGAAATCAATATGATCGTTTCTGACATCGAAGCTAACGCCCT                                                                                                                                                                                                                                                               >  1:19676/1‑301`  
                                                     `tgtataagagacagCACTAAAGGAGACACACCATGTTCAAACTGATTAAGAAGTTAGGACAACTGCTGGTTCGTATGTACAACGTGGAAGCCAAGCGACTGAACGATGAGGCGCGTAAAGAGGCCACACAGTCACGCGCTCTGGCGATTCGCTCCAACGAACTGGCTGACAGTGCATCCACTAAAGTTACCGAGGCTGCCCGTGTGGCAAACCAAGCTCAACAGCTTTCCAAATTCTTTGAGTAATCAAACAGGAGAAATCAATATGATCGTTTCTGACACCGAAGCTAACGCCCTCTTAG                                                                                                                                                                                                                                                          <  1:79039/287‑1`  
                                                     `ATTCTAATACGACTCACTAAAGGAGAAACACCATGTTCAAACTGATTAAGAAGTTAGGCCAACTGCTGGTTCGTATGTACAACGTGGAAGCCAAGCGACTGAACGATGAGGCTCGTAAAGAGGCCACACAGTCACGCGCTCTGGCGATTCGCTCCAACGAACTGGCTGACAGTGCATCCACTAAAGTTACCGAGGCTGCCCGTGTGGCAAACCAAGCTCAACAGCTTTCCAAATTCTTTGAGTAATCAAACAGGAGAAACCATTATGATCGTTTCTGACATCGAAGCTAACGCCCTCTTAG                                                                                                                                                                                                                                                          <  1:271267/301‑1`  
                                                      `TTCTAATACGACTCACTAAAGGAGACACACCATGTTCAAACTGATTAAGAAGTTAGGCCAACTGCTGGTTCGTATGTACAACGTGGAAGCCAAGCTACTGAACGATGAGGATCGTAAAGAGGCCACACAGTCACGCGCTCTGGCGATTCGCTCCAACGAACTGGCTGACAGTGCATCCACTAAAGTTACCGAGGCTGCCCGTGTGGCAAACCAAGCTCAACAGCTTTCCAAATTCTTTTAGTAATCAAACAGGAGAAACCATTATGATCGTTTCTGACCTCCAAGCTACCGCCCTCTTAta                                                                                                                                                                                                                                                         >  1:167388/1‑299`  
                                                       `tgtgtataagagacagAAAGGAGACACACCATGTTCAAACTGATTAAGAAGTTAGGCCAACTGCTGGTTCGTATGTACAACGTGGAAGCCAAGCGACTGAACGATGAGGCTCGTAAAGAGGCCACACAGTCACGCGCTCTGGCGATTCGCTCCAACGAACTGGCTGACAGTGCATCCACTAAAGTTACCGAGGCTGCCCGTGTGGCAAACCAAGCTCAACAGCTTTCCAAATTCTTTGAGTAATCAAACAGGAGAAATCAATATGATCGTTTCTGACATCGAAGCTAACGCCCTCTTAGAG                                                                                                                                                                                                                                                        <  1:205072/285‑1`  
                                                       `tataagagacagCACTAAAGGAGACACACCATGTTCAAACTGATTAAGAAGTTAGGCCAACTGCTGGTTCGTATGTACAACGTGGAAGCCAAGCGACTGAACGATGAGGCTCGTAAAGAGGCCACACAGTCACGCGCTCTGGCGATTCGCTCCAACGAACTGGCTGACAGTGCATCCACTAAAGTTACCTAGGCTGCCCGTGTGGCAAACCAAGCTCAACAGCTTTCCAAATTCTTTGAGTAATCAAACAGGAGAAACCATTATGATCGTTTCTGACATCGAAGCCAACGCCCTCTTAGAG                                                                                                                                                                                                                                                        <  1:191840/289‑1`  
                                                       `TCTAATACGACTCACTAAATTAGACACACCATGTTCAAACTGATTAAGAAGTTAGGCCAACTGCTGGTTCGTATGTACAACGTGGAAGCCAAGCGACTTAACGATGAGGCTCGTAAAGAGGCCACACAGTCACGCGCTCTGGCGATTCGCTCCAACGAACTGGCTTACAGTGCATCCACTAAAGTTACCGAGGCTGCCCGTGTGGCAAACCAAGCTCAACAGCTTCCCAAATTCTTTGAGTAATCAAACAGGAGAAACCATTATGATCGTTTCTGACATCGAAGCTAACGCCCTCTTAGAG                                                                                                                                                                                                                                                        <  1:266995/301‑1`  
                                                       `TCTAATACGACTCACTAAAGGAGACACACCATGTTCAAACTGATTAAGAAGTTAGGCCAACTGCTGGTTCGTATGTACAACGTGGAAGCCAAGCGACTGAACGATGAGGCTCGTAAAGAGGCCACACAGTCACGCGCTCTGGCGATTCGCTCCAACGAACTGGATGACAGTGCATCCACTAAAGTTACCGAGGCTGCCCGTGTGGCAAACCAAGCTCAACAGCTTTCCAAATTCTTTGAGTAATCAAACAGGAGAAACCATTATGATCGTTTCTGACATCGAAGCTAACGCCCTCTTAGAG                                                                                                                                                                                                                                                        <  1:115670/301‑1`  
                                                         `TAATACTACTCACTAAAGGAGACACACCATTTTCAAACTTATTAAGAGGTCAGGCCAACTGCGGGTTCGTATGTACAACGTGGAAGCCAAGCGACTGAACGATGAGGCTCGTAAAGAGGCCACACAGTCACGCGCTCTGGCGATTCGCTCCAACGAACTGGCTGACAGTGCATCCACTAAAGTTACCGAGGCTGCCCGTGTGGCAAACCAAGCTCAACAGCTTTCCAAATTCTTTGAGTAATCAAACAGGAGAAATCAATATGATCGTTTCTGACATCGAAGCTAACGCCCTCTTAGAGAG                                                                                                                                                                                                                                                      <  1:117373/301‑1`  
                                                          `aagagacagCACTAAAGGAGACACACCATGTTCAAACTGATTAAGAATTTAGGCCAACTGCTGGTTCGTATGTACAACGTGGAAGCCAAGCGACTGAACGATGAGGCTCGTAAAGAGGCCACACAGTCACGCGCTCTGGCGATTCGCTCCAACGAACTGGCTGACAGTGCATCCACTAAAGTTACCGAGGCTGCCCGTGTGGCAAACCAAGCTCAACAGCTTTCCAAATTCTTTGAGTAATCAAACAGGAGAAACCATTATGATCGTTTCTGACATCGAAGCCAACGCCCTCTTAGAGAGC                                                                                                                                                                                                                                                     <  1:67650/292‑1`  
                                                               `GACTCACTAAAGGAGACACACCATGTTCAAACTGATTAAGAAGTTAGGCCAACTGCTGGTTCGTATGTACAACGTGGAAGCCAAGCGACTGAACGATGAGGCTCGTAAAGAGGCCACACAGTCACGCGCTCTGGCGATTCGCTCCAACGAACTGGCTGACAGTGCATCCACTAAAGTTACCGAGGCTGCCCGTGTGGCAAACCAAGCTCAACAGCTTTCCAAATTCTTTGAGTAATCAAACAGGAGAAATCAATATGATCGTTTCTGACATCGAAGCTAACGCCCTATTAGAGAGCGTCAC                                                                                                                                                                                                                                                <  1:328615/301‑1`  
                                                                   `CACTAAAGGAGACACACCATGTTCAAACTGATTAAGAAGTTAGGCCAACTGCTGGTTCGTATGTACAACGTGGAAGCCAAGCGACTGAACGATGAGGCTCGTAAAGAGGCCACACAGTCACGCGCTCTGGCGATTCGCTCCAACGAACTGGCTGACAGTGCATCCACTAAAGTTACCGAGGCTGCCCGTGTGGCAAAACAAGCTCAACAGCTTTCCAAATTCTTTGAGTAATCAAACAGGAGAAACCATTATGATCGTTTCTGACATCGAAGCTAACGCCCTCTTAGAGAGCGTCACTAAG                                                                                                                                                                                                                                            >  1:48713/1‑301`  
                                                                       `AAAGGAGACACACCATGTTCAAACTGATTAAGAAGTTAGGCCAACTGCTGGTTCGTATGTACAACGTGGAAGCCAAGCGACTGAACGATGAGGCTCGTAAAGAGGCCACACAGTCACGCGCTATGGCGATTCGCTCCAACGAACTGGCTGACAGTGCATCCACTAAAGTTACCGAGGCTGCCCGTGTGGCAAACCAAGCTCAACAGCTTTCCAAATTCTTTGAGTAATCAAACAGGAGAAACCATTATGATCGTTTCTGACATCGAAGCCAACGCCCTCTTAGAGAGCGTCACTAAGTTCC                                                                                                                                                                                                                                        <  1:89602/301‑1`  
                                                                         `agacagcccatCATGTTCAAACTGATTAAGAAGTTAGGCCAACTGCTGGTTCGTATGTACAACGTGGAAGCCAAGCGACTGAACGATGAGGCTCGTAAAGAGGCCACACAGTCACGCGCTCTGGCGATTCGCTCCAACGAACTGGCTGACAGTGCATCCACTAAAGTTACCGAGGCTGCCCGTGTGGCAAACCAAGCTCAACAGCTTTCCAAATTCTTTGAGTAATCAAACAGGAGAAATCAATATGATCGTTTCTGACATCGAAGCTAACGCCCTCTTAGAGAGCGTCACTAAGTTCCAC                                                                                                                                                                                                                                      <  1:208946/290‑1`  
                                                                         `AGGAGACAAACCATGATCAAACTGATTAAGAAGTTAGGCCAACAGCTGGTTCGTATGTACAACGTGGAAGCCAAGCGACTGAACGATGAGGCTCGTAAAGAGGCCACACAGTCACGCGCTCTGGCGATTCGCTCCAACGAACTGGCTGACAGTGCATCCACTAAAGTTACCGAGGCTGCCCGTGTGGCAAAACAAGCTCAACAGCTTTCCAAATTCTTTGAGTAATCAAACAGGAGAAATCAATATGATCGTTTCTGACATCGAAGCTAACGCCCTCTTAGAGAGCGTCACTAAGTTCCAC                                                                                                                                                                                                                                      <  1:254660/301‑1`  
                                                                         `AGGAAAAACACCATGTTCAAACTGATTTAGAAAATAGGTCAAATGCATGTTCGTAAGTACAACGTTGAAGCCAAGCGAAGGAACGATGAGGCTCGTAAAGAGGCCACACAGTCACGCGCTATGGCAATTCGCTCCAACGAACGGGCTGAAAGTGCATCCATTAAAGTTACCGAGGCTGCCCGTGTGGCAAACCAAGGTCAACAGCTTTCCAAATTCCTTTAGTAATCAAAAAGGAGAAATCAATATTATCGTGTCTGACATCGAAGCTAACGCTCTCTTAGAGAGCGTCACTAAGTTCCAC                                                                                                                                                                                                                                      <  1:19867/301‑1`  
                                                                                `ACACCATGTTCAAACTGATTGAGAAGTTAGGCCAACTGCTGGTTCGTATGTACAACGTGGAAGCCAAGCGACTGAACGATGAGGCTCGTAAAGAGGACACACAGTCACGCGCTCTGGCGATTCGCTCCAACGAACTGGCTGACAGTGCAACCACTAAAGTTACCGAGGCTGCCCGTGTGGCAAACCAAGCTCAACAGCTTTCCAACTTCTTTGAGTAATCAAACAGGAGAAACCAATATGATCGTTTCTGACATCGAAGCCAACGCCCTCTTAGAGAGCGTCACTAAGTTCCACTGCGGGG                                                                                                                                                                                                                               <  1:122259/301‑1`  
                                                                                `ACACCATGTTCAAACTGATTAAGAAGTTAGGCCAACTGCTGGTTCGTATGTACAACGTGGAAGCCAAGCGACTGAACGATGAGGCTCGTAAAGAGGCCACACAGTCACGCGCTCTGGCGATTCGCTCCAACGAACTGGCTGACAGTGCATCCACTAAAGTTACCGAGGCTGCCCGTGTGGCAAACCAAGCTCAACAGCTTTCCAAATTCTTTGAGTAATCAAACAGGAGAAATCAATATGATCGTTTCTGACATCGAAGCTAACGCCCTCTTAGAGAGCGTCACTAAGTTCCACTGCGGGG                                                                                                                                                                                                                               <  1:284451/301‑1`  
                                                                                 `CACCATGTTCAAACTGATTAAGAAGTTAGGCCAACTGCTGGTTCGTATGTACAACGTGGAAGCCAAGCGACTGAACGATGAGGCTCGTAAAGAGGCCACACAGTCACGCGCTCTGGCGATTCGCTCCAACGAACTGGCTGACAGTGCATCCACTAAAGTTACCGAGGCTGCCCGTGTGGCAAACCAAGCTCAACAGCTTTCCAAATTCTTTGAGTAATCAAACAGGAGAAATCAATATGATCGTTTCTGACATCGAAGCCAACGCCCTCTTAGAGAGCGTCACTAAGTTCCACTGCGGGGT                                                                                                                                                                                                                              <  1:2060/301‑1`  
                                                                                 `CACCATGTTCAAACTGATTAAGAAGTTAGGCCAACTGCTGGTTCGTATGTACAACGTGGAAGCCAAGCGACTGAACGATGAGGCTCGTAAAGAGGCCACACAGTCACGCGCTCTGGCGATTCGCTCCAACGAACTGGCTGACAGTGCATCCACTAAAGTTACCGAGGCTGCCCGTGTGGCAAACCAAGCTCAACAGCTTTCCAAATTCTTTGAGTAATCAAACAGGAGAAACCATTATGATCGTTTCTGACATCGAAGCTAACGCCCTCTTAGAGAGCGTCACTAAGTTCCACTGCGGGGT                                                                                                                                                                                                                              >  1:130304/1‑301`  
                                                                                 `CACCATGTTCAAACTGATTAAGAAGTTAGGCCAACTGCTGGTTCGTATGTACAACGTGGAAGCCAAGCGACTGAACGATGAGGCTCGTAAAGAGGCCACACAGTCACGCGCTCTGGCGATTCGCTCCAACGAACTGGCTGACAGTGCATCCACTAAAGTTACCGAGGCTGCCCGTGTGGCAAACCAAGCTCAACAGCTTTCCAAATTCTTTGAGTAATCAAACAGGAGAAACCATTATGATCGTTTCTGACATCGAAGCTAACGACCTCTTAGAGctgtctcttatacacatctccgagcc                                                                                                                                                                                                                              >  1:215405/1‑275`  
                                                                                  `ACCATGTTCAAACTGATTAAGAAGTTAGGCCAACTGCTGGTTCGTATGTACAACGTGGAAGCCAAGCGACTGAACGATGAGGCTCGTAAAGAGGCCACACAGTCACGCGCTCTGGCGATTCGCTCCAACGAACTGGCTGACAGTGCATCCACTAAAGTTACCGAGGCTGCCCGTGTGGCAAACCAAGCTCAACAGCTTTCCAAATTCTTTGAGTAATCAAACAGGAGAAACCATTATGATCGTTTCTGACATCGAAGCTAACGCCCTCTTAGAGAGCGTCACTAAGTTCCACTGCGGGGTT                                                                                                                                                                                                                             >  1:223291/1‑301`  
                                                                                           `AAACTGATTAAGAAGTTATGCCAACTGCTGGTTCGTATGTACAACGTGGAAGCCAAGCGACTGAACGATGAGGCTCGTAAAGAGGCCACACAGTCACGCGCTCTGGCGATTCGCTCCAACGAACTGGCTGACAGTGCATCCACTAAAGTTACCGAGGCTGCCCGTGTGGCAAACCAAGCTAAACAGCTTTCCAAATTCTTTGAGTAATCAAACAGGAGAAACCATTATGATCGTTTCTGACATCGAAGCTAACGCCCTCTTAGAGAGCGTCACTAAGTTCCACTGCGGGGTTATCTACGAC                                                                                                                                                                                                                    <  1:44049/301‑1`  
                                                                                           `AAACTGATTAAGAAGTTAGGCCAACTGCTGGTTCGTATGTACAACGTGGAAGCCAAGCGACTGAACGATGAGGCTCGTAAAGAGGCCACACAGTCACGCGCTCTGGCGATTCGCTCCAACGAACTGGCTGACAGTGCATCCACTAAAGTTACCGAGGCTGCCCGTGTGGCAAACCAAGCTCAACAGCTTTCCAAATTCTTTGAGTAATCAAACAGGAGAAACCATTATGATCGTTTCTGACATCGAAGCTAACGCCCTCTTAGAGAGCGTCACTAAGTTCCACTGCGGGGTTATCTACGAC                                                                                                                                                                                                                    <  1:221183/301‑1`  
                                                                                                 `ATTAAGAAGTTAGGCCAACTGCTGGTTCGTATGTACAACGTGGAAGCCAAGCGACTGAACGATGAGGCTCGTAAAGAGGCCACACAGTCACGCGCTCTGGCGATTCGCTCCAACGAACTGGCTGACAGTGCATCCACTAAAGTTACCGAGGCTGCCCGTGTGGCAAACCAAGCTCAACAGCTTTCCAAATTCTTTGAGTAATCAAACAGGAGAAACCATTATGATCGTTTCTGACATCGAAGCTAACGCCCTCTTAGAGAGCGTCACTAAGTTCCACTGCGGGGTTATCTTCGACTACTCC                                                                                                                                                                                                              >  1:149155/1‑301`  
                                                                                                      `aAAGTTAGGCCAACTGCTGGTTCGTATGTACAACGTGGAAGCCAAGCGACTGAACGATGAGGCTCGTAAAGAGGCCACACAGTCACGCGCTCTGGCGATTCGCTCCAACGAACTGGCTGACAGTGCATCCACTAAAGTTACCGAGGCTGCCCGTGTGGCAAACCAAGCTCAACAGCTTTCCAAATTCTTTGAGTAATCAAACAGGAGAAATCAATATGATCGTTTCTGACATCGAAGCTAACGCCCTCTTAGAGAGCGTCACTAAGTTCCACTGCGGGGTTATCTACGACTACTCCACCGC                                                                                                                                                                                                         <  1:12178/300‑1`  
                                                                                                             `GGCCAACTGCTGGTGCGTATGTACAACGTGGAAGCCAAGCGACTGAACGATGTGGCTCGTAAAGAGGCCACACAGTCACGCGCTCTGGCGATTCGCTCCAACGAACTGGCTGACAGTGCATCCACTAAAGTTACCGAGGCTGCCCGTGTGGCAAACCAAGCTCAACAGCTTTCCAAATTCTTTGAGTAATCAAACAGGAGAAATCAATATGATCGTTTCTGACATCGAAGCTAACGCCCTCTTAGAGAGCGTCACTAAGTTCCACTGCGGGGTTATCTACGACTACTCCACCGCTGAGTAC                                                                                                                                                                                                  <  1:314542/301‑1`  
                                                                                                                  `ACTGCTGGTTCGTATGTACAACGTGGAAGCCAAGCGACTGAACGATGAGGCTCGTAAAGAGGCCACACAGGCACGTGCTCTGGCGATTCGCTCCAACGAACTGGCTGACAGTGCATCAACTAAAGTTACCGAGGCTGCCCGTGTGGCAAACCAAGCTCAACAGCTTTCCAAATTCTTTGAGTAATCAAACAGGAGAAATCAATATGATCGTTTCTGACATCGAAGCTAACGCCCTCTCAGAGAGCGTCACTACGTTCCACTGCGGGGTTATCTACGACTACTCCACCGCTGAGTACGTAAG                                                                                                                                                                                             >  1:163502/1‑301`  
                                                                                                                     `GCTGGTTCGTATGTACAACGGGGAAGCCAAGCGACTGAACGATGAGGCTCGTAAAGAGGCCACACAGTCACGCGCTCTGGCGATTCGCTACAACGAACTGGCTGACAGTGCATCCACTAAAGTTACCGAGGCTGCCCGTGTGGCAAACCAAGCTCAACAGCTTTCCAAATTCTTTGAGTAATC‑AATAGGAGAAATCAATATGATCGTTTCTGACATCGAAGCTAACGCCCTCTTAGAGAGCGTCACTAAGTTCCACTGCGGGGTTATCTACGACTACTCCACCGCTGAGTACGTAAGCTAC                                                                                                                                                                                         <  1:104702/301‑1`  
                                                                                                                          `TTCGTATGTACAACGTGGAAGCCAAGCGACTGAACGATGAGGATCGTAAAGAGGCCACACAGTCACGCGCTCTGGCGATTCGCTCCAACGAACTGGCTGACAGTGCATCCACTCAAGTTACCGAGGCTGCCCGTGTGGCAAACCAAGCTCAACAGCTTTCCAAATTCTTTGATTAATCAAACAGGAGAAATCAATATGATCGTTTCTGCCATCGAAACTAACGCCCTCTTAGAGATCGTCCCTAAGTTCCACTGCGGGGTTATCTACGACTACTCCACCGCTGCGTACGTAAGCTACCGTC                                                                                                                                                                                     >  1:46111/1‑301`  
                                                                                                                                     `aaaggGGAAGCCAAGCGACTGAACGAGGAGGCTCGTAAAGAGGCAACACAGGCACGCGCTCTGGCGATTCGCGCCAACGAACTGGCTGACAGTGCATCCACTAAAGTTACCGAGGCTGCCCGTGAGGCAAACCAAGCGCAACAGCTTTCCAAATTCTTTGAGTAATCAAACAGGAGAAATCAATATGATCGTTTCTGACATCGAAGCTAACGCACTCTTAGAGAGCGTCACTAAGTTCCACTGCGGGGTTATCTACGACTACTCCACCGCTGAGTACGTAAGCTACCGTACGAGTGACTTC                                                                                                                                                                          <  1:130837/296‑1`  
                                                                                                                                          `ggcAGCCAAGCGACTGAACGATTAGGCTCATAAAGAGGCCACACAGTCACGCGCTCTGGCGATTCGCTCCAACGAACTGGCTGACAGTGCATCCACTAAAGTTACCGAGGCTGCCCGTGTGGCAAACCAAGCTCAACAGCTTTCCAAATTCTTTGAGTAATC‑AATAGTAGAAATCAATATGATTGTTTCTGACATCGAAGCTAACGCCCTCTTAGAGAGCGTCACTAAGTTCCACTGCGGGGTTATCTACGACTACTCCACCGCTGAGTACGTAAGCGACCGTCCGAGTGACTTCGGTGCG                                                                                                                                                                    <  1:308959/298‑1`  
                                                                                                                                                 `cAGCGACTGAACGATGAGGCTCGTAAAGAGGCCACACAGTCACGCGCTCTGGCGATTCGCTCCAACGAACTGGCTGACAGTGCATCCACTAAAGTTACCGATGCTGCCCGTGTGGCAAACCAAGCTCAACAGCTTTCCAAATTCTTTGAGTAATCAAACAGGAGAAATCAATATGATCGTTTCTGACATCGAAGCTAACGCCCTCTTAGAGAGCGTCACTAAGTTCCACTGCGGGGTTATCTACGACTACTCCACCGCTGAGTACGTAAGCTACCGTCCGAGTGACTTCGGTGCGTATCTG                                                                                                                                                              <  1:323116/300‑1`  
                                                                                                                                                   `GCGACTGAACGATGAGGCTCGTAAAGAGGCCACACAGTCACGCGCTCTGGCGATTCGCTCCAACGAACTGGCTGACAGTGCATCCACTAAAGTTACCGAGGCTGCCCGTGTGGCAAACCAAGCTCAACAGCTTTCCAAATTCTTTGAGTAATCAAACAGGAGAAATTAATATGATCGTTTCTGACATCGAAGCTAACGCCCTCTTAGAGAGCGTCACTAAGTTCCACTGCGGGGTTATCTACGACTACTCCACCGCTGAGTACGTAAGCTACCGTCCGAGTGACTTCGGTGCGTATCTGGA                                                                                                                                                            <  1:233295/301‑1`  
                                                                                                                                                   `GCGACTGAACGATGAGGCTCGGAAAGAGGCCACACAGTCACGCGCTCTGGCGATTCGCTCCAACGAACTGGCTGACAGTGCATCCACTAAAGTTACCGAGGCTGCCCGTGTGGCAAACCAAGCTCAACAGCTTTCCAAATTCTTTGAGTAATCAAACAGGAGAAATCAATATGATCGTTTCTGACATCGAAGCTAACGCCCTCTTAGAGAGCGTCACTAAGTTCCACTGCGGGGTTATCTACGACTACTCCACCGCTGAGTACGTAAGCTACCGTCCGAGTGACTTCGGTGCGTATCGGGA                                                                                                                                                            <  1:127317/301‑1`  
                                                                                                                                                    `CGACTGAACGATGAGGCTCGTAAAGAGGCCACACAGTCACGCGCTCTGGCGATTCGCTCCAACGAACTGGCTGACAGTGCATCCACTAAAGTTACCGAGGCTGCCCGTGTGGCAAACCAAGCTCAACAGCTTTCCAAATTCTTTGAGTAATCAAACAGGAGAAACCATTATGATCGTTTCTGACATCGAAGCTAACGCCCTCTTAGAGAGCGTCACTAAGTTCCACTGCGGGGTTATCTACGACTACTCCACCGCTGAGTACGTAAGCTACCGTCCGAGTGACTTCGGTGCGTATCTGGAT                                                                                                                                                           <  1:238945/301‑1`  
                                                                                                                                                             `GATGAGGCTCGTAAAGAGGCCACACAGTCACGCGCTCTGGCGATTCGCTCCAACGAACTGGCTGACAGTGCATCCACTAAAGTTACCGAGGCTGCCCGTGTGGCAAACCAAGCTCAACAGCTTTCCAAATTCTTTGAGTAATCAAACAGGAGAAATCAATATGATCGTTTCTGACATCGAAGCTAACGCCCTCTTAGAGAGCGTCACTAAGTTCCACTGCGGGGTTATCTACGACTACTCCACCGCTGAGTACGTAAGCTACCGTCCGAGTGACTTCGGTGCGTATCTGGATGCGCTGGAA                                                                                                                                                  <  1:243258/301‑1`  
                                                                                                                                                                   `GCTCGTAAAGAGGCCACACAGTCACGCGCTCTGGCGATTCGCTCCAACTAACTGGCTGACAGTGCATCCACTAAAGTTACCGAGGCTGCCCGTGTGGCAAACCAAGCTCAACAGCTTTCCAAATTCTTTTAGTAATCAAACAGGAGAAATCAATATGATCGTTTCTGACATCGAAGCTAACGCCCTCTTAGAGAGCGTCACTAAGTTCCACTGCTGGGTTATCTACGACTACTCCACCGCTGAGTACGTAAGCTACCGTCCGAGTGACTTCGGTGCGTATCTGGATGCGCTGGAAGCCGAG                                                                                                                                            >  1:29957/1‑301`  
                                                                                                                                                                             `AGGCCACACAGTCACGCGCTCTGGCGATTCGCTCCAACGAACTGGCTGACAGTGCATCCACTAAAGTTACCGAGGCTGCCCGTGTGGCAAACCAAGCTCAACAGCTTTCCAAATTCTTTGAGTAATCAAACAGGAGAAACCATTATGATCGTTTCTGACATCGAAGCTAACGCCCTCTTAGAGAGCGTCACTAAGTTCCACTGCGGGGTTATCTACGACTACTCCACCGCTGAGTACGTAAGCTACCGTCCGAGTGACTTCGGTGCGTATCTGGATGCGCTGctgtctcttatacacatct                                                                                                                                  >  1:295000/1‑282`  
                                                                                                                                                                                       `GTCACTCGCTCTGGCGATTCGCTCCAACGAACTGGCTGACAGTGCATCCACTAAAGTTACCGAGGCTGCCCGTGTGGCAAACCAAGCTCAACAGCTTTCCAAATTCTTTGAGTAATCAAACAGGAGAAATCAATATGATCGTTTCTGACATCGAAGCTAACGCCCTCTTAGAGAGCGTCACTAAGTTCCACTGCGGGGTTATCTACGACTACTCCACCGCTGAGTACGTAAGCTACCGTCCGAGTGACTTCGGTGCGTATCTGGATGCGCTGGAAGCCGAGGTTGCACGAGGCGGTCTTAT                                                                                                                        >  1:195884/1‑301`  
                                                                                                                                                                                         `CACGCGCTCTGGCGATTCGTTCCAACGAACGGGCTGACAGTGCATCCACTAAAGTTACCGAGGCTGCCCGTGTGGCAAACCAAGCTCAACAGCTTTCCAAATTCTTTGAGTAATCAAACAGGAGGAACCATTATGATCGTTTCTGACATCGAAGCTAACGCCCTCTTAGAGAGCGTCACTAAGTTCCACTGCGGGGTTATCTACGACTACTCCACCGCTGAGTACGTAAGCTACCGTCCGAGTAACTTCGGTGCGTATCTGGATGCGCTGGAAGCCGAGGTTGCACGAGGCGGTCTTATTG                                                                                                                      <  1:334305/301‑1`  
                                                                                                                                                                                         `CACGCGCTCTGGCGATTCGCTCCACCGAACTGGCTGACAGTGCATCCACTAAAGTTACCGAGGCTGCCCGTGTGGCAAACCAAGCTCAACAGCTTTCCAAATTCTTTGAGTAATCAAACAGGAGAAATCAATATGATCGTTTCTGACATCGAAGCTAACGCCATCTTAGAGAGCGTCACTAAGTTCCACTGCGGGGTTATCTACGACTACTCCACCGCTGAGTACGTAAGCTACCGTCCGAGTGACTTCGGTGCGTATCTGGATGCGCTGGAAGCCGAGGTTGCACGAGGCGGTCTTATTG                                                                                                                      <  1:50137/301‑1`  
                                                                                                                                                                                           `CGCGCTCTGGCGATTCGCTCCAACGAACTGGCTGACAGTGCATCCACTAAAGTTACCGAGGCTGCCCGTGTGGCAAACCAAGCTCAACAGCTTTCCAAATTCTTTGAGTAATC‑AATAGGAGAAATCAATATGATCGTTTCTGACATCGAAGCTAACGCCCTCTTAGAGAGCGTCACTAAGTTCCACTGCGGGGTTATCTACGACTACTCCACCGCTGAGTACGTAAGCTACCGTCCGAGTGACTTCGGTGCGTATCTGGATGCGCTGGAAGCCGAGGTTGCACGAGGCGGTCTTATTGTGT                                                                                                                   <  1:6542/301‑1`  
                                                                                                                                                                                                   `GGCGATTCGCTCCAACGAACTGGCTGACAGTGCATCCACTAAAGTTACCGAGGCTGCCCGTGTGGCAAACCAAGCTCAACAGCTTTCCAAATTCTTTGAGTAATCAAACAGGAGAAATCAATATGATCGTTTCTGACATCTAAGCTAACGCCCTCTTAGAGAGCGTCACTAAGTTCCACTGCTGGGTTATCTACGACTACTCCACCGCTGAGTACGTAAGCTACCGTCCGAGTGACTTCGGTGCGTATCTGGATGCGCTGGAAGCCGAGGTTGCACGAGGCGGTCTTATTGTGTTCCACAA                                                                                                            <  1:70053/301‑1`  
                                                                                                                                                                                                   `GGCGATTCGCTCCAACGAACTGGCTGACAGTGCATCCACTAAAGTTACCGAGGCTGCCCGTGTGGCAAACCAAGCTCAACAGCTTTCCAAATTCTTTGAGTAATCAAACAGGAGAAATCAATATGATCGTTTCTGACATCGAAGCTAACGCCCTCTTAGAGAGCGTCACTAAGTTCCACTGCGGGGTTATCTACGACTACTCCACCGCTGAGTACGTAAGCTACCGTCCGAGTGACTTCGGTGCGTATCTGGATGCGCTGGAAGCCGAGGTTGCACGAGGCGGTCTTATTGTGTTCCACAA                                                                                                            <  1:112871/301‑1`  
                                                                                                                                                                                                                 `gagacagGGCTGACAGTGCATCCACTAAAGTTACCGAGGCTGCCCGTGTGGCAAACCAAGCTCAACAGCTTTCCAAATTCTTTGAGTAATC‑AATAGGAGAAATCAATATGATCGTTTCTGACATCGAAGCTAACGCCCTCTTAGAGAGCGTCACTAAGTTCCACTGCGGGGTTATCTACGGCTACTCCACCGCTGAGTACGTAAGCTACCGTCCGAGTGACTTCGGTGCGTATCTGGATGCGCTGGAAGCCGAGGTTGCACGAGGCGGTCTTATTGTGTTCCACAACGGTCACAAGTATGA                                                                                             <  1:307807/294‑1`  
                                                                                                                                                                                                                    `AACTGGCTGACAGTGCATCCACTAAAGTTACCGAGGCTGCACGTGTGGCAAACCAAGCTCAACAGCTTTCCAAATTCTTTGAGTAATCAAACAGGAGAAACCATTATGATCGTTTCTGACATCGAAGCTAACGCCCTCTTAGAGAGCGTCACTAAGTTCCACTGCGGGGTTATCTACGACTACTCCACCGCTGAGTACGTAAGCTACCGTCCGAGTGACTTCGGTGCGTATCTGGATGCGCTGGAAGCCGAGGTTGCACGAGGCGGTCTTATTGTGTTCCACAACGGTCACAAGTATGACG                                                                                           <  1:270132/301‑1`  
                                                                                                                                                                                                                          `ctgaacGTGCATCCACTAAAGTTACCGAGGCTGCCCGTGTGGCAAACCAAGCTCAACAGCTTTCCAAATTCTTTGAGTAATCAAACAGGAGAAATCAATATGATCGTTTCTGACATCGAAGCTAACGCCCTCTTAGAGAGCGTCACTAAGTTCCACTGCGGGGTTATCTACGACTACTCCACCGCTGAGTACGTAAGCTACCGTCCGAGTGACTTCGGTGCGTATCTGGATGCGCTGGAAGCCGAGGTTGCACGAGGCGGTCTTATTGTGTTCCACAACGGTCACAAGTATGACGTTCCTG                                                                                     <  1:39071/295‑1`  
                                                                                                                                                                                                                                      `CCACTAAAGTTACCGAGGCTGCCCGTGTGGCAAACCAAGCTCAACAGCTTTCCAAATTCTTTGAGTAATCAAACAGGAGAAACCATTATGATCGTTTCTGACATCGAAGCTAACGCCCTCTTAGAGAGCGTCACTAAGTTCCACTGCGGGGTTATCTACGACTACTCCACCGCTTAGTACGTAAGCTACCGTCTGAGTGACTTCGGTGCGTATCTGGATGCGCTGGAAGCCGAGGTTGCACGAGGCGGTCTT‑TTGTGTTCTACAACGGACACAAGTATGACGTTCCTGCATTTACCAAACT                                                                        >  1:178104/1‑301`  
                                                                                                                                                                                                                                       `CACTAAAGTTACCGAGGCTGCCCGTGTGGCAAACCAAGCTCAACAGCTTTCCAAATTCTTTGAGTAATCAAACAGGAGAAACCATTATGATCGTTTCTGACATCGAAGCTAACGCCCTCTTAGAGAGCGTCACTAAGTTCCACTGCGGGGTTATCTACGACTACTCCACCGCTGAGTACGTAAGCTACCGTCCGAGTGACTTCGGTGCGTATCTGGATGCGCTGGAAGCCGAGGTTGCACGAGGCGGTCTTATTGTGTTCCACAACGGTCACAAGTATGACGTTCCTGCATTGACCAAACT                                                                        >  1:60343/1‑301`  
                                                                                                                                                                                                                                            `AAGTGACCGAGGCTGCCCTTGTGGCAAACCAAGCTCAACAGCTTTCCAAATTCTTTGAGTAATCAAACAGGAGAAACCATTATGATCGTTTCTGACATCGAAGCCAACGCCCTCTTAGAGAGCGTCACTAAGTTCCACTGCGGGGTTATCTACGACTACTCCACCGCTGAGTACGTAAGCTACCGTCCGAGTGACTTCGGTGCGTATCTGGATGCGCTGGAAGCCGAGGTTGCACGTGGCGGTCTTATTGTGTTCCACAACGGTCACAAGTATGACGTTCCTGCATTGACCAAACTGGCAA                                                                   <  1:9335/301‑1`  
                                                                                                                                                                                                                                                           `CCCGTGTGGCAAACCAAGCTCAACAGCTTTCCAAATTCTTTGAGTAATC‑AATAGGAGAAATCAATATGATCGTTTCTGACATCGAAGCTAACGCCCTCTTAGAGAGCGTCACTAAGTTCCACTGCGGTGTTATCTACGACTACTCCACCGCTGAGTACGTAAGCTACCGTCCGAGTGACTTCGGTGCGTATCTGGATGCGCTGGAAGCCGAGGTTGCACGAGGCGGTCTTATTGTGTTCCACAACGGTCACAAGTATGACGTTCCTTCATTGACCAAACTGGCAAAGTTGCAATTGAACCG                                                   <  1:62422/301‑1`  
                                                                                                                                                                                                                                                                         `CAAGCTCAACAGCTTTCCAAATTCTTTGAGTAATCAAACAGGAGAAATCAATATGATCGTTTCTGACATCGAAGCTAACGCCCTCTTAGAGAGCGTCACTAAGTTCCACTGCGGGGTTATCTACGACTACTCCACCGCTGAGTACGTAAGCTACCGTCCGAGTGACTTCGGTGCGTAGCTGGATGCGCTGGAAGCCGAGGTTGCACGAGGCGGTCTTATTGTGTTCCACAACGGCCACAAGTATGACGTTCCTGCATTGACCAAACTGGCAAAGTTGCAATTGAACCGAGAGTTCCACCTT                                      <  1:226258/301‑1`  
                                                                                                                                                                                                                                                                          `AAGCTCAACAGCTTTCCAAATGCTTTGAGTAAGCAAACAGGAGAAATCAATATGATCGTTTCTGACATCGAAGCTAACGCCCTCTTAGAGAGCGTCACTAAGTTACACTGCGGGGTTATCTACGACTACTCCACCGCTGAGTACGTAAGCTACCGTCCGAGTGACTTCGGTGCGTATATGGATGCGCTGGAAGCCGAGGTTGCACGAGGCGGTCTTATTGTGTTCCACAACGGTCACAAGTATGACGTTCCTGCATTGACCAAACTGGCAAAGTTGCAATTGAACCGAGAGTTCCACCTTC                                     <  1:10213/301‑1`  
                                                                                                                                                                                                                                                                            `GCTCAACAGCTTTCCAAATTCTTTGAGTAATC‑AATAGGAGAAATCAATATGATCGTTTCTGACATCGAAGCTAACGCCCTCTTAGAGAGCGTCACTAAGTTCCACTGCGGGGTTATCTACGACTACTCCACCGCTTAGTACGTAAGCTACCGTCCGAGTGACGTCGGTGCGTATCTGGATGCGCTGGAAGCCGAGGTTGCACGAGGCGGTCTTATTGTGTTCCACAACGGTCACAAGTATGACGTTCCTGCATTGACCAAACTGGCAAAGTTGCAATTGAACCGAGAGTTCCACATTCCTC                                  >  1:286253/1‑301`  
                                                                                                                                                                                                                                                                             `CTCAACAGCTTTCCAAATTCTTTGAGTAATCAAACAGGAGAAATCAATATGATCGTTTCTGACATCGAAGCTAACGCCCTCTTAGAGAGCGTCACTAAGTTCCACTGCGGGGTTATCTACGACTACTCCACCGCTGAATACGTAAGCTACCGTCCGAGTGACTTCGGTGCGTATCTGGACGCGCTGGAAGCCGAGGTTGCACGAGGCGGTCTTATTGTGTTCCACAACGGTCACAAGTATGACGTTCCTGCATTGACCAAACTGGCAAAGTTGCCATTGAACCGAGAGTTCCCCCTTCCTC                                  >  1:140822/1‑301`  
                                                                                                                                                                                                                                                                                 `tcgggaatggttaaaagggacagAATA‑AATAGGAGAAATCAATATGATCGTTTCTGACATCGAAGCTAACGCCCTCTTAGAGAGCGTCACTAAGTTCCACTGCGTGGTTACCTACGACTACTCCACCGCTGAGTACGTAAGCTACCGTCCGAGTGACTTCGGTGCGTATCTGGATGCGCTGGAAGCCGAGGTTGCACTAGGCGGTCTTATTGTGTTCCACAACGGTCACAAGTATGACGTTCCTGCATTGACCAAACTGGCAAAGTTGCAATTGAACCGAGAGTTCCACCTTCCTCGTGAG                             <  1:239611/278‑1`  
                                                                                                                                                                                                                                                                                 `ACAGCTTTCCAAATTCTTTGAGTAATC‑AATAGGAGAAATCAATATGATCGTTTCTGACATCTAAGCTAACGCCCTCTTAGAGAGCGTCACTAAGTTCCACTGCGGGGTTATCTACGACTACTCCACCGCTGAGTACGTAAGCTACCGTCCGAGTGACTTCGGTGCGTATCTGGATGCGCTGGAAGCCGAGGTTGCACGAGGCGGTCTTATTGTGTTCCACAACGGTCACAAGTATGACGTTCCTGCATTGACCAAACTGGCAAAGTTGCAATTGAACCGAGAGTTCCACCTTCCTCGTGAG                             <  1:87924/301‑1`  
                                                                                                                                                                                                                                                                                     `tcggagatgggtataagagacagaAATAGGAGAAATCAATAGGATCGTTTCTGACATCGAAGCTAACGCCCTCTTAGAGAGCGTAACTAAGTTCCACTGCGGGGTTATCTACGACTACTCCACCGCTGAGTACGTAAGCTACAGTCCGGGTGACTTCGGTGCGTATCTGGATGCGCTGGAAGCCGAGGTTGCACGAGGCGGTCTTATTGTGTTCCACAACGGTCACAAGTATGACGTTCCTGCATTGACCAAACTGGCAAAGTTGCAATTGAACCGAGAGTTCAACCTTCCTCGTGAGAAC                          <  1:26515/277‑1`  
                                                                                                                                                                                                                                                                                       `TTCCAAATTCTTTGAGTAATCAAACAGGAGAAACCATTATGATCGTTTCTGACATCGAAGCTAACGCCCTCTTAGAGAGCGTCACTAAGTTCCACTGCGGGGTTATCTACGACTACTCCACCGCTGAGTACGTAAGCTACCGTCCGAGTGACTTCGGTGCGTATCTGGATGCGCTGGAAGCCGAGGTTGCACGAGGCGGTCTTATTGTGTTACACAACGGTCACAAGTATGACGTTCCTGCATTGACCAAACTGGCAAAGTTGCAATTGAACAGAGAGTTCCACCTTACTCGTGAGAACTG                        >  1:156732/1‑301`  
                                                                                                                                                                                                                                                                                        `TCCAAATTCTTTGAGTAATCAAACAGGAGAAATCAATATGATCGTTTCTGACATCGAAGCTAACGCCCTCTTAGAGAGCGTCACTAAGTTCCACTGCGGGGTTATCTACGACTACTCCACCGCTGAGTACGTAAGCTACCGTCCGAGTGACTTCGGTGCGTATCTGGATGCGCTGGAAGCCGAGGTTGCACGAGGCGGTCTTATTGTGTTCCACAACGGTCACAAGTATGACGTTCCTGCATTTACCAAACTGGCCAAGTTGCAATTTAACCGAGAGTTCCACCTTCCCCGTGAGAACTGT                       >  1:323387/1‑301`  
                                                                                                                                                                                                                                                                                          `CAAATTCTTTGAGTAATCAAACAGGAGAAACCATTATGATCGTTTCTGACATCGAAGCTAACGCCCTCTTAGAGAGCGTCACTAAGTTCCACTGCGGGGTTATCTACGACTACTCCACCGCTGAGTACGTAAGCTACCGTCCGAGTGACTTCGGTGCGTATCTGGATGCGCTGGAAGCCGAGGTTGCACGAGGCGGTCTTATTGTGTTCCACAACGGTCACAAGTATGACGTTCCTGCATTGACCAAACTGGCAAAGTTGCAATTGAACCGAGAGTTCCACCTTCCTAGTGAGAACTGTAT                     <  1:136638/301‑1`  
                                                                                                                                                                                                                                                                                              `TTCTTTGAGTAATCAAACAGGAGAAACCATTATGATCGTTTCTGACATCGAAGCTAACGCCCTCTTAGAGAGCGTCACTAAGTTCCACTGCGGGGTTATCTACGACTACTCCACCGCTTAGTACGTAAGCTTCCGTCCTAGTGACTTCGGTGCGTATCTGGATGCGCTGGAAGCCGAGGTTGCACGAGGCGGTCTTATTGTGTTCTACAACGGTCACAAGTATGACTTTCCTGCATTGACCAAACTGGCAAAGTTTCAATTGAACCGAGAGTTCCACCTTCCTCGTTAGActgtctcttat                 >  1:327117/1‑290`  
                                                                                                                                                                                                                                                                                              `TTCTTTGAGTAATCAAACAGGAGAAACCATTATGATCGTTTCTGACATCGAAGCTAACGCCCTCTTAGAGAGCGTCACTAAGTTCCACTGCGGGGTTATCTACGACTACTCCACCGCTGAGTACGTAAGCTACCGTCCGAGTGACTTCGGTGCGTATCTGGATGCGCTGGAAGCCGAGGTTGCACGAGGCGGTCTTATTGTGTTCCACAACGGTCACAAGTATGACGTTCCTGCATTGACCAAACTGGCAAAGTTGCAATTGAACCGAGAGTTCCACCTTCCTCGTGctgtctcttatacc                 >  1:340260/1‑287`  
                                                                                                                                                                                                                                                                                                `aagagacagATC‑AATAGGAGAAATCAATATGATCGTTTCTGACATCGAAGCTAACGCCCTCTTAGAGAGCGTCACTAAGTTCCACTGCGTGGTTATCTACGACTACTCCACCGCTGAGTACGTAAGCTACCGTCCGAGTGACTTCGGTGCGTATCTGGATGCGCTGGAAGCCGAGGTTGCACGAGGCGGTCTTATTGTGTTCCACAACGGTCACAAGTATGACGTTCCTGCATTGACCAAACTGGCAAAGTTGCAATTGAACCGAGAGTTCCACCTTCCTCGTGAGAACTGTATTGACACC              <  1:152587/292‑1`  
                                                                                                                                                                                                                                                                                                      `GTAATCAAACAGGAGAAACCATTATGATCGTTTCTGACATCGAAGCTAACGCCCTCTTAGAGAGCGTCACTAAGTTCCACTGCGGGGTTATCTACGACTACTCCACCGCTGAGTACGTAAGCTACCGTCCGAGTGACTTCGGTGCGTATCTGGATGCGCTGGAAGCCGAGGTTGCACGAGGCGGTCTTATTGTGTTCCACAACGGTCACAAGTATGACGTTCCTGCATTGACCAAACTGGCAAAGTTGCAATTGAACCGAGAGTTCCACCTTCCTCGTGAGAACTGTATTGACACtgtctc         >  1:242713/1‑295`  
                                                                                                                                                                                                                                                                                                       `aAATC‑AATAGGAGAAATCAATATGATCGTGTCTGACATCGAAGCTAACGCCCTCTTAGAGAGCGTCACTAAGTTCCACTGCGGGGTTATCTACGACTACTCCACCGCTGAGTACGTAAGCTACCGTCCGAGTGACTTCGGTGCGTATCTGGATGCGCTGGAAGCCGAGGTTGCACGAGGCGGTCTTATTGTGTTCCACAACGGTCACAAGTATGACGTTCCTGCATTGACCAAACTGGCAAAGTTGCAATTGAACCGAGAGTTCCACCTTCCTCGTGAGAACTGTATTGACACCCTTGTGT       <  1:310771/300‑1`  
                                                                                                                                                                                                                                                                                                       `TAATC‑AATAGGAGAAATCAATATGATCGTTTCTGACATCGAAGCTAACGCCCTCTTAGAGAGCGTCACTAAGTTCCACTGCGGGGTTATCTACGACTACTCCACCGCTGAGTACGTAAGCTACCGTCCGAGTGACTTCGGTGCGTATCTGGATGCGCTGGAAGTCGAGGTTGCACGAGGCGGTCTTATTGTGTTCCACAACGGTCACAAGTATGACGTTCCTGCATTGACCAAACTGGCAAAGTTGCAATTGAACCGAGAGTTCCACCTTCCTCGTGAGAACTGTATTGACACCCTTGTGT       <  1:137657/301‑1`  
                                                                                                                                                                                                                                                                                                       `TAATC‑AATAGGAGAAATCAATATGATCGTTTCTGACATCGAAGCTAACGCCCTCTTAGAGAGCGTCACTAAGTTCCACTGCGGGGTTATCTACGACTACTCCACCGCTGAGTACGTAAGCTACCGTCCGAGTGACTTCGGTGCGTATCTGGATGCGCTGGAAGCCGAGGTTGCACGAGGCGGTCTTATTGTGTTCCACAACGGTCACAAGTATGACGTTCCTGCATTGACCAAACTGGCAAAGTTGCAATTGAACCGAGAGTTCCACCTTCCTCGTGAGAACTGTATTGACACCCTTGTGT       <  1:203392/301‑1`  
                                                                                                                                                                                                                                                                                                       `TAATC‑AATAGGAGAAATCAATATGATCGTTTCTGACATCGAAGCTAACGCCCTCTTAGAGAGCGTCACTAAGTTCAACTGCGGGGTTATCTACGACTACTCCACCGCTGAGTACGTAAGCTACCGTCCGAGTGACTTCGGTGCGTATCTGGATGCGCTGGAAGCCGAGGTTGCACGAGGCGGTCTTATTGTGTTCCACAACGGTCACAAGTATGACGTTCCTGCATTGACCAAACTGGCAAAGTTGCAATTGAACCGAGAGTTCCACCTTCCTCGTGAGAACTGTATTGACACCCTTGTGT       <  1:160234/301‑1`  
                                                                                                                                                                                                                                                                                                        `AATC‑AATAGGATAAATCAATATGATCGTTTCTGACATCGAAGCTAACGCCCTCTTAGAGAGCGTCACTAAGTTCCACTGCGGGGTTATCTACGACTACTCCACCGCTGAGTACGTAAGCTACCGTCCGAGTTACTTCGGTGCGTATCTGGATGCGCTGGAAGCCGAGGTTGCACGAGGCGGTCTTATTGTGTTCCACAACGGTCACAAGTATGACGTTCCTGCATTGACCAAACTGGCACAGTTGCAATTGAACCGAGAGTTCCACCTTCCTCGTGAGAACTGTATTGACACCCTTGTGTT      >  1:34938/1‑301`  
                                                                                                                                                                                                                                                                                                        `AATC‑AATAGGAGAAATCAATATGATCGTTTCTGACATCGAAGCTAACGCCCTCTTAGAGAGCGTCACTAAGTTCCACTGCGGGGTTATCTACGACTACTCCACCGCTGAGTACGTAAGCTACCGTCCGAGTGACTTCGGTTCGTATCTGCATGAGCTGGAAGCCGAGGTTGCACGAGGCGGTCTTATTGTGTTCAACAACGGTCACAAGTATGACGTTCCTGCATTGACCAAACTGGCAACGTTGCAATTGAACCGAGAGTTCCACCTTCCTCGTGAGAACTGTATTGACACCCTTGTGTT      >  1:24169/1‑301`  
                                                                                                                                                                                                                                                                                                        `AATC‑AATAGGAGAAATCAATATGATCGTTTCTGACATCGAAGCTAACGCCCTCTTAGAGAGCGTCACTAAGTTCCACTGCGGGGTTATCTACGACTACTCCACCGCTGAGTACGTAAGCTACCGTCCGAGTGACTTCGGTGCGTATCTGGATGCGCTGGAAGCCGAGGTTGCACGAGGCGGTCTTATTGTGTTCCACAACGGTCACAAGTATGACGTTCCTGCATTGACCAAACTGGCAAAGTTGCAATTGAACCGAGAGTTCCACCTTCCTCGTGAGAACTGTATTGACctgtctcttat      >  1:184334/1‑290`  
                                                                                                                                                                                                                                                                                                        `AATC‑AATAGGAGAAATCAATATGATCGTTTCTGACATCGAAGCTAACGCCCTCTTAGAGAGCGTCACTAAGTTCCACTGCGGGGTTATCTACGACTACTCCACCGCTGAGTACGTAAGCTACCGTCCGAGTGACTTCGGTGCGTATCTGGATGCGCTGGAAGCCGAGGTTGCACGAGGCGGTCTTATTGTGTTCCACAACGGTCACAAGTATGACGTTCCTGCATTGACCAAACTGGCAAAGTTGCAATTGAACCGAGAGTTCCACCTTCCTCGTGAGAACTGTATTGACACCCTTGTGTT      >  1:123217/1‑301`  
                                                                                                                                                                                                                                                                                                        `AATC‑AATAGGAGAAATCAATATGATCGTTTCTGACATCGAAGCTAACGCCCTCTTAGAGAGCGTCACTAAGTTCCACTGCGGGGTTATCTACGACTACTCAACCGCTGAGTACGTAAGCTACCGTCCGAGTGACTTCGGTGCGTATCTGGATGCGCTGGAAGCCGAGGTTGCACGAGGCGGTCTTATTGTGTTCCACAACGGTCACAAGTATGACGTTCCTGCATTGACCAAACTGGCAAAGTTGCAATTGAACCGAGAGTTCCACCTTCCTCGTGAGctgtctcttatacacatctccga      >  1:129877/1‑278`  
                                                                                                                                                                                                                                                                                                         `ATC‑AATAGGAGAAATCAATATGATCGTTTCTGACATCGAATCTAACGCCCTCTTAGAGAGCGTCACTAAGTTCCACTGCGGGGTTATCTACGACTACTCCACCGCTGAGTACGTAAGCTACCGTCCGAGTGACTTCGGTGCGTATCTGGATGCGCTGGAAGCCGAGGTTGCACGAGGCGGTCTTATTGTGTTACACAACGGTCACAAGTATGACGTTCCTGCATTGACCAAACTGGCAAAGTTGCAATTGAACCGAGAGTTCCACCTTCCTCGTGAGAACTGTATTGACACCCTTGTGTTG     <  1:212123/301‑1`  
                                                                                                                                                                                                                                                                                                         `ATC‑AATAGGAGAAATCAATATGATCGTTTCTGACATCGAAGCTAACGCCCTCTTAGAGAGCGTCACTAAGTTCCACTGCGGGGTTATCTACGACTACTCCACCGCTGAGTACGTAAGCTACCGTCCGAGTGACTTCGGTGCGTATCTGGATGCGCTGGAAGCCGAGGTTTCACGAGGCGGTCTTATTGTGTTCCACAACGGTCACAAGTATGACGTTCCTGCATTGTCCAAACTGGCAAAGTTGCAATTGAACCGAGAGTTCCACCTTCCTCGTGAGAACTGTATTGACACCCTTGTGTTt     >  1:280609/1‑300`  
                                                                                                                                                                                                                                                                                                         `ATC‑AATAGGAGAAATCAATATGATCGTTTCTGACATCGAAGCTAACGCCCTCTTAGAGAGCGTCACTAAGTTCCACTGCGGGGTTATCTACGACTACTCCACCGCTGAGTACGTAAGCTACCGTCCGAGTGACTTCGGTGCGTATCTGGATGCGCTGGAAGCCGAGGTTGCACGAGGCGGTCTTATTGTGTTCCACAACTGTCACAAGTATGACGTTCCTGCATTGACCAAACTGGCAAAGTTGCAATTGAACCGAGAGTTCCACCTTCCTCGTGAGAACTGTATTGACACCCTTGTGTTG     >  1:8859/1‑301`  
                                                                                                                                                                                                                                                                                                         `ATC‑AATAGGAGAAATCAATATGATCGTTTCTGACATCGAAGCTAACGCCCTCGTAGAGAGCGTCACTAAGTTCCACTGCGGGGTTATCTACGACTACTCCACAGCTGAGTACGTAAGCTACCGTCCGAGTGACTTCGGTGCGTATCTGGATGCGCTGGAAGCCGAGGTTGCACGAGGCGGTCTTATTGTGTTCCACAACGGTCACAAGTATGACGTTCCTGCATTGACCAAACTGGCAAAGTTGCAATTGAACCGAGAGTTCCACCTTCCTCGTGAGAACTGTATTGACACCCTTGTGTTG     <  1:5945/301‑1`  
                                                                                                                                                                                                                                                                                                         `ATC‑AATAGGAGAAATCAATATGATCGTTTCTGACATCGAAGCCAACGCCTGCTTAGAGAGCGTCACTAAGTTCCACTGCGGGGTTATCTACGACTACTCCACCGCTGAGTACGTAAGCTACCGTCCGAGTGACTTCGGTGCGTATCTGGATGCGCTGGAAGCCGAGGTTGCACGAGGCGGTCTTATTGTGTTCCACAACGGTCACAAGTATGACGTTCCTGCATTGACCAAACTGGCAAAGTTGCAATTGAACCGAGAGTTCCACCTTCCTCGTGAGAACTGTATTGACACCCTTGTGTTG     <  1:39248/301‑1`  
                                                                                                                                                                                                                                                                                                          `TC‑AATAGGAGAAATCAATATGATCGTTTCTGACATCGAAGCTAACGCCCTCTTAGAGAGCGTCACTAAGTTCCACTGCGGGGTTATCTACGACTACTCCACCGCTGAGTACGTAAGCTACCGTCCGAGTGACTTCGGTGCGTATCTGGATGCGCTGGAAGCCGAGGTTGCACGAGGCGGTCTTATTGTGTTCCACAACGGTCACAAGTATGACGTTCCTGCATTGACCAAACTGGCAAAGTTGCAATTGAACCGAGAGTTCCACCTTCCTCGTGAGAACTGTATTTTCACCCTTGTGTctg    >  1:245884/1‑298`  
                                                                                                                                                                                                                                                                                                          `TC‑AATAGGAGAAATCAATATGATCGTTTCTGACATCGAAGCTAACGCCCTCTTAGAGAGCGTCACTAAGTTCCACTGCGGGGTTATCTACGACTACTCCACCGCTGAGTACGTAAGCTACCGTCCGAGTGACTTCGGTGCGTATCTGGATGCGCTGGAAGCCGAGGTTGCACGAGGCGGTCTTATTGTGTTCCACAACGGTCACAAGTATGACGTTCCTGCATTGACCAAACTGGCAAAGTTGCAATTGAACCGAGAGTTCCACCTTCCTCGTGAGAACTGTATTGACACCCTTGTGTctg    >  1:86046/1‑298`  
                                                                                                                                                                                                                                                                                                           `C‑AATAGGAGAAATCAATATGATCGTTTCTGACATCGAAGCTAACGCCCTCTTAGAGAGCGTCACTAAGTTCCACTGCGGGGTTATCTACGACTACTCCACCGCTGAGTACGTACGCTACCGTCCGAGTGACTTCGGTGCGTATCTGGATGCGCTGGAAGCCGAGGTTGCACGAGGCGGTCTTATTGTGTTCCACAACGGTCACAAGTTTGACGTTCCTGCATTGACCAAACTGGCAAAGTTGCAATTGAACCGAGAGTTCCACCTTCCTCGTGAGAATTGTTTTGACACTCTTGTGTTGTC   >  1:55488/1‑301`  
                                                                                                                                                                                                                                                                                                           `C‑AATAGGAGAAATCAATATGATCGTTTCTGACATCGAAGCTAACGCCCTCTTAGAGAGCGTCACTAAGTTCCACTGCGGGGTTATCTACGACTACTCCACCGCTGAGTACGTAAGCTACCGTCCGAGTGACTTCGGTTCGTATCTGGATGCGCTGGAAGCCGAGGTTGCACGAGGCGGTCTTATTGTGTTCCACAACGGTCACAAGTATGACGTTCCTGCATTGACCAAACTGGCAAAGTTGCAATTGAACCGAGAGTTCCACCTTCCTCGTGAGAACTGTATTGACACtgtctcttataa   >  1:289560/1‑289`  
                                                                                                                                                                                                                                                                                                           `C‑AATAGGAGAAATCAATATGATCGTTTCTGACATCGAAGCTAACGCCCTCTTAGAGAGCGTCACTAAGTTCCACTGCGGGGTTATCTACGACTACTCCACCGCTGAGTACGTAAGCTACCGTCCGAGTGACTTCGGTGCGTATCTGGATGCGCTGGAAGCCGAGGTTGCACGAGGCGGTCTTATTGTGTTCCACAACGGTCACAAGTATGACGTTCCTGCATTGACCAACCTGGCAAAGTTGCAATTGAACCGAGAGTTCCACCTTCCTCGTGAGAACTGTATTGATACCCTTGTTTTGTt   >  1:201741/1‑300`  
                                                                                                                                                                                                                                                                                                           `C‑AATAGGAGAAATCAATATGATCGTTTCTGACATCGAAGCTAACGCCCTCTTAGAGAGCGTCACTAAGTTCCACTGCGGGGTTATCTACGACTACTCCACCGCTGAGTACGTAAGCTACCGTCCGAGTGACTTCGGTGCGTATCTGGATGCGCTGGAAGCCGAGGTTGCACGAGGCGGTCTTATTGTGTTCCACAACGGTCACAAGTATGACGTTCCTGCATTGACCAAACTGGCAAAGTTGCAATTGAACCGAGAGTTCCACCTTCCTCGTGAGAACTGTATTGACACtgtctcttatac   >  1:2290/1‑289`  
                                                                                                                                                                                                                                                                                                           `C‑AATAGGAGAAATCAATATGATCGTTTCTGACATCGAAGCTAACGCCCTCTTAGAGAGCGTCACTAAGTTCCACTGCGGGGTTATCTACGACTACTCCACCGCTGAGTACGTAAGCTACCGTCCGAGTGACTTCGGTGCGTATCTGGATGCGCTGGAAGCCGAGGTTGCACGAGGCGGTCTTATTGTGTTCCACAACGGTCACAAGTATGACGTTCCTGCATTGACCAAACTGGCAAAGTTGCAATTGAACCGAGAGTTCCACCTTCCTCGTGAGAACTGTATTGACACCCTTGTGTctgt   >  1:314049/1‑297`  
                                                                                                                                                                                                                                                                                                           `C‑AATAGGAGAAATCAATATGATCGTTTCTGACATCGAAGCTAACGCCCTCTTAGAGAGCGTCACTAAGTTCCACTGCGGGGTTATCTACGACTACTCCACCGCTGAGTACGTAAGCTACCGTCCGAGTGACTTCGGTGCGTATCTGGATGCGCTGGAAGCCGAGGTTGCACGAGGCGGTCTTATTGTGTTCCACAACGGTCACAAGTATGACGTTCCTGCATTGACCAAACTGGCAAAGTTGCAATTGAACCGAGAGTTCCACCTTCCTCGTGAGAACTGTATTGACACCCTTGTGTTGTC   >  1:36365/1‑301`  
                                                                                                                                                                                                                                                                                                           `C‑AATAGGAGAAATCAATATGATCGTTTCTGACATCGAAGCTAACGCCCTCTTAGAGAGCGTCACTAAGTTCCACTGCGGGGTTATCTACGACTACTCCACCGCTGAGTACGTAAGCTACCGTCCGAGTGACTTCGGTGCGTATCTGGATGCGCTGGAAGCCGAGGTTGCACGAGGCGGTCTTATTGTGTTCCACAACGGTCACAAGTATGACGTTCCTGCATTGACCAAACTGGCAAAGTTGCAATTGAACCGAGAGTTCCACCTTCCTCGTGAGAACTGTATTGACACCCTTGTGTTGTC   >  1:224579/1‑301`  
                                                                                                                                                                                                                                                                                                           `C‑AATAGGAGAAATCAATATGATCGTTTCTGACATCGAAGCTAACGCCCTCTTAGAGAGCGTCACTAAGTTCCACTGCGGGGTTATCTACGACTACTCCACCGCTGAGTACGTAAGCTACCGTCCGAGTGACTTCGGTGCGTATCTGGATGCGCTGGAAGCCGAGGTTGCACGAGGCGGTCTTATTGTGTTCCACAACGGTCACAAGTATGACGTTCCTGCATTGACCAAACTGGCAAAGTTGCAATTGAACCGAGAGTTCCACCTTCCTCGTGAGAACTGTATTGACACCCTGGTGTTGTC   >  1:248425/1‑301`  
                                                                                                                                                                                                                                                                                                           `C‑AATAGGAGAAATCAATATGATCGTTTCTGACATCGAAGCTAACGCCCTCTTAGAGAGCGTCACTAAGTTCCACTGCGGGGTTATCTACGACTACTCCACCGCTGAGTACGTAAGCTACCGTCCGAGTGACTTCGGTGCGTATCTGGATGCGCTGGAAGCCGAGGTTGCACGAGGCGGTCTTATTGTGTTCCACAACGGTCACAAGTATGACGTTCCTGCATTGACCAAACTGGCAAAGTTGCAATTGAACCGAAAGTTCCACCTTCCTCGTGAGAACTGTATTGACACCCCTGTctctta   >  1:318452/1‑295`  
                                                                                                                                                                                                                                                                                                           `C‑AATAGGAGAAATCAATATGATCGTTTCTGACATCGAAGCTAACGCCCTCTTAGAGAGCGTCACTAAGTTCCACTGCGGGGTTATCTACGACTACTCCACCGCTGAGTACGTAAGCTACCGTCCGAGTGACTTCGGTGCGTATCTGGATGCGCTGGAAGCCGAGGTTGCACGAGGCGGTCTTATTGTGTTCCACAACGGTCACAAGTATGACGTTCCTGCATTGACCAAACTGGCAAAGTTGCAATTGAACAGAGAGTTCCACCTTCCTCGTGAGAACTGTATTGACACCCTTGTGTTGTt   >  1:51425/1‑300`  
                                                                                                                                                                                                                                                                                                           `C‑AATAGGAGAAATCAATATGATCGTTTCTGACATCGAAGCTAACGCCCTCTTAGAGAGCGTCACTAAGTTCCACTGCGGGGTTATCTACGACTACTCCACCGCTGAGTACGTAAGCTACCGTCAGAGTGACTTCGGTGCGTATCTGGATGCGTTGGAAGCCGAGGTTGCACGAGGCGGTCTTATTGTGTTCCACAACGGTCACAAGTATGACGTTCCTGCATTGACCAAACTGGCAAAGTTGCAATTGAACCGAGAGTTCCACCTTCCTCGTGAGAACTGCATTGACACCCTTGTGTTGTC   >  1:151038/1‑301`  
                                                                                                                                                                                                                                                                                                           `C‑AATAGGAGAAATCAATATGATCGTTTCTGACATCGAAGCCAACGCCCTCTTAGAGAGCGTCACTAAGTTCCACTGCGGGGTTATCTACGACTACTCCACCGCTGAGTACGTAAGCTACCGTCCGAGTGACTTCGGTGCGTATCTGGATGCGCTGGAAGCCGAGGTTGCACGAGGCGGTCTTATTGTGTTCCACAACGGTCACAAGTATGACGTTCCTGCATTGACCAAACTGGCAAAGTTGCAATTGAACCGAGAGTTCCACCTTCCTCGTGAGAACTGTATTGACctgtctcttataca   >  1:42550/1‑287`  
                                                                                                                                                                                                                                                                                                             `AATAGGAGAAATCAATATGATCGTTTCTGACATCGAAGCCAACGCCCTCGTAGAGAGCGTCACTAAGTTCCCCTGCGGGGTTATCTACGACTACTCCACCGCTGAGTACGTAAGCTACCGTCCGAGTGACTTCGGTGCGTATCTGGATGCGCTGGAAGCCGAGGTTGCACGAGGCGGTCTTATTGTGTTCCACAACGGTCACAAGTATGACGTTCCTGCATTGACCAAACTGGCAAAGTTGCAATTGAACCGAGAGTTCCACCTTCCTCGTGAGAACTGTATTGACACCCTTGTGTTGTCA  <  1:251176/301‑1`  

                                                                                                                                                                                                                                                                                                                                                                                                                                                                                                                                                                                                                            
`AGGATTCTTGATGACTTTCCAGACGACTACGAGAAGTTTCGCTGGAGAGTCCCATTCTAATACGACTCACTAAAGGAGACACACCATGTTCAAACTGATTAAGAAGTTAGGCCAACTGCTGGTTCGTATGTACAACGTGGAAGCCAAGCGACTGAACGATGAGGCTCGTAAAGAGGCCACACAGTCACGCGCTCTGGCGATTCGCTCCAACGAACTGGCTGACAGTGCATCCACTAAAGTTACCGAGGCTGCCCGTGTGGCAAACCAAGCTCAACAGCTTTCCAAATTCTTTGAGTAATC‑AA‑‑‑‑‑‑‑‑‑‑‑‑‑‑‑‑‑‑‑‑‑‑‑‑‑‑‑‑‑‑‑‑‑‑‑‑‑‑‑‑‑‑‑‑‑‑‑‑‑‑‑‑‑‑‑‑‑‑‑‑‑‑‑‑‑‑‑‑‑‑‑‑‑‑‑‑‑‑‑‑‑‑‑‑‑‑‑‑‑‑‑‑‑‑‑‑‑‑‑‑‑‑‑‑‑‑‑‑‑‑‑‑‑‑‑‑‑‑‑‑‑‑‑‑‑‑‑‑‑‑‑‑‑‑‑‑‑‑‑‑‑‑‑‑‑‑‑‑‑‑‑‑‑‑‑‑‑‑‑‑‑‑‑‑‑‑‑‑‑‑‑‑‑‑‑‑‑‑‑‑‑‑‑‑‑‑‑‑‑‑‑‑‑‑‑‑‑‑‑‑‑‑‑‑‑‑‑‑‑‑‑‑‑‑‑‑‑‑‑‑‑‑‑‑‑‑‑‑‑‑‑‑‑‑‑‑‑‑‑‑‑‑‑‑‑‑‑‑‑‑‑‑‑‑‑‑‑‑‑‑‑‑‑‑‑‑‑‑‑‑‑‑‑‑‑‑‑‑‑‑‑‑‑‑‑‑‑‑‑‑‑‑‑‑‑‑‑‑‑  >  NC_001604.1/13267‑13568`  
`‑‑‑‑‑‑‑‑‑‑‑‑‑‑‑‑‑‑‑‑‑‑‑‑‑‑‑‑‑‑‑‑‑‑‑‑‑‑‑‑‑‑‑‑‑‑‑‑‑‑‑‑‑‑‑‑‑‑‑‑‑‑‑‑‑‑‑‑‑‑‑‑‑‑‑‑‑‑‑‑‑‑‑‑‑‑‑‑‑‑‑‑‑‑‑‑‑‑‑‑‑‑‑‑‑‑‑‑‑‑‑‑‑‑‑‑‑‑‑‑‑‑‑‑‑‑‑‑‑‑‑‑‑‑‑‑‑‑‑‑‑‑‑‑‑‑‑‑‑‑‑‑‑‑‑‑‑‑‑‑‑‑‑‑‑‑‑‑‑‑‑‑‑‑‑‑‑‑‑‑‑‑‑‑‑‑‑‑‑‑‑‑‑‑‑‑‑‑‑‑‑‑‑‑‑‑‑‑‑‑‑‑‑‑‑‑‑‑‑‑‑‑‑‑‑‑‑‑‑‑‑‑‑‑‑‑‑‑‑‑‑‑‑‑‑‑‑‑‑‑‑‑‑‑‑‑‑‑‑‑‑‑‑‑‑‑‑‑‑‑‑‑‑‑‑‑‑‑‑‑‑‑‑‑‑‑‑‑‑‑‑‑‑‑‑taatc‑aaTAGGAGAAATCAATATGATCGTTTCTGACATCGAAGCTAACGCCCTCTTAGAGAGCGTCACTAAGTTCCACTGCGGGGTTATCTACGACTACTCCACCGCTGAGTACGTAAGCTACCGTCCGAGTGACTTCGGTGCGTATCTGGATGCGCTGGAAGCCGAGGTTGCACGAGGCGGTCTTATTGTGTTCCACAACGGTCACAAGTATGACGTTCCTGCATTGACCAAACTGGCAAAGTTGCAATTGAACCGAGAGTTCCACCTTCCTCGTGAGAACTGTATTGACACCCTTGTGTTGTCA  >  NC_001604.1/14339‑14637`

  

Alignment Legend

Aligned base mismatch/match (shaded by quality score): `ATCG/ATCG` `< 3 ≤` `ATCG/ATCG` `< 11 ≤` `ATCG/ATCG` `< 24 ≤` `ATCG/ATCG` `< 36 ≤` `ATCG/ATCG` `< 38 ≤` `ATCG/ATCG`

Unaligned base: `atcg`    Masked matching base: `atcg`    Alignment gap: `‑`    Deleted base: `‑`

Reads not counted as support for junction

read\_name Not counted due to insufficient overlap past the breakpoint.

read\_name Not counted due to not crossing MOB target site duplication.
